# A comparison of two procedures for verbal response time fractionation

**DOI:** 10.3389/fpsyg.2014.01213

**Published:** 2014-10-24

**Authors:** Lotje van der Linden, Stéphanie K. Riès, Thierry Legou, Borís Burle, Nicole Malfait, F.-Xavier Alario

**Affiliations:** ^1^Laboratoire de Psychologie Cognitive-UMR 7290, Centre National de la Recherche Scientifique (CNRS), Aix Marseille UniversitéMarseille, France; ^2^Department of Psychology, Helen Wills Neuroscience Institute, University of CaliforniaBerkeley, CA, USA; ^3^Laboratoire Parole et Langage-UMR 7309, Centre National de la Recherche Scientifique (CNRS), Aix Marseille UniversitéAix-en-Provence, France; ^4^Laboratoire de Neurosciences Cognitives-UMR 7291, Centre National de la Recherche Scientifique (CNRS), Aix Marseille UniversitéMarseille, France; ^5^Institut de Neurosciences de la Timone-UMR 7289, Centre National de la Recherche Scientifique (CNRS), Aix Marseille UniversitéMarseille, France

**Keywords:** mental chronometry, speech production, motor control, articulation, electromyography (EMG), psycholinguistics

## Abstract

To describe the mental architecture between stimulus and response, cognitive models often divide the stimulus-response (SR) interval into stages or modules. Predictions derived from such models are typically tested by focusing on the moment of response emission, through the analysis of response time (RT) distributions. To go beyond the single response event, we recently proposed a method to fractionate verbal RTs into two physiologically defined intervals that are assumed to reflect different processing stages. The analysis of the durations of these intervals can be used to study the interaction between cognitive and motor processing during speech production. Our method is inspired by studies on decision making that used manual responses, in which RTs were fractionated into a premotor time (PMT), assumed to reflect cognitive processing, and a motor time (MT), assumed to reflect motor processing. In these studies, surface EMG activity was recorded from participants' response fingers. EMG onsets, reflecting the initiation of a motor response, were used as the point of fractionation. We adapted this method to speech-production research by measuring verbal responses in combination with EMG activity from facial muscles involved in articulation. However, in contrast to button-press tasks, the complex task of producing speech often resulted in multiple EMG bursts within the SR interval. This observation forced us to decide how to operationalize the point of fractionation: as the first EMG burst after stimulus onset (the stimulus-locked approach), or as the EMG burst that is coupled to the vocal response (the response-locked approach). The point of fractionation has direct consequences on how much of the overall task effect is captured by either interval. Therefore, the purpose of the current paper was to compare both onset-detection procedures in order to make an informed decision about which of the two is preferable. We concluded in favor or the response-locked approach.

## Introduction

Conveying a verbal message requires cognitive as well as motor processing. Firstly, cognitive processing is required to mentally represent the intended message, to select the appropriate words from lexicon, and to retrieve the words' syntactic, phonological, and phonetic properties (Levelt et al., 1999). In turn, motor processing is required to articulate the utterance overtly. This complex physical action involves moving more than 100 muscles simultaneously (Meister et al., 2007). Yet, despite the fact that both cognitive and motor processes are necessary for conveying a spoken message, they are typically investigated in isolation rather than in combination (i.e., in the field of psycholinguistics, cf. Dell, 1986; Levelt et al., 1999; vs. the fields of phonology, cf. Browman and Goldstein, 1992; and motor control, cf. Guenther, 2006, respectively). This separation likely stems from the common assumption that the transition from cognitive to motor processing occurs in an entirely serial (discrete) fashion, such that articulation can only be initiated after cognitive processing has finished (Levelt et al., 1999). Notably, in his original paper on the (non-serial) cascade model, McClelland (1979), argued that response execution may be a discrete event, of which the duration does not depend on previous processing: “The cascade model (…) shares with the discrete stage model the assumption that the execution of a response is a discrete event. However, in the discrete stage model only one process is at work at a time, whereas in the cascade model, all processes except response execution are at work all of the time” (McClelland, 1979, p. 291).

Recently, it has been suggested that this view is an (over)simplification. Several studies demonstrated that some effects of incomplete cognitive processing (e.g., partially activated phonological representations) do “cascade down” to articulatory processing (e.g., Hennessey and Kirsner, 1999; Kello et al., 2000; Goldrick and Blumstein, 2006; McMillan and Corley, 2010). For example, Goldrick and Blumstein (2006) asked participants to produce “tongue twisters.” Participants had to rapidly repeat aloud, for example, the syllables “keff geff geff keff.” Next, the researchers compared the acoustic signals of erroneous and correct responses. The results showed that erroneously produced syllables contained traces of the intended target, as if two phonemes had been prepared simultaneously. The authors interpreted this as cascading activation from cognitive to articulatory processing (Goldrick and Blumstein, 2006). These and related findings show that psycholinguistic and motor-control research should be combined to obtain a complete understanding of speech production (Hickok, 2014 and commentaries).

### RT fractionation in studies on decision making using manual responses

In previous work, we proposed a novel method to shed further light on the relationship between cognitive and articulatory processes of speech production (Riès et al., 2012, 2014). The basic principle of our method is mental chronometry, an approach that is often used in the field of decision making using manual responses. In such studies, overall response times (RTs) are fractionated into premotor times (PMTs), assumed to reflect cognitive processing, and motor times (MT's), assumed to reflect motor processing (e.g., Botwinick and Thompson, 1966; Possamaĩ et al., 2002). RT fractionation is highly suitable for investigating the relationship between cognitive and motor processing, because serial and cascaded models make different predictions about the effect of task manipulations on the duration of both intervals. On the one hand, serial models assume that a motor response can only be initiated and executed when cognitive processing is complete. Following this logic, a cognitive manipulation should only influence (i.e., lengthen or shorten, depending on the condition) the duration of PMTs (cognitive processing), whereas the duration of MTs (motor processing) should be unaffected (i.e., constant across conditions). On the other hand, modern versions of cascaded models assume that it is possible to initiate response execution on the basis of partial information, before cognitive processing is complete. The fact that cognitive and motor processes are concurrent opens the possibility that a cognitive manipulation influences the duration of both PMT and MT intervals.

For example, Possamaĩ et al. (2002) carried out a response-selection task in which participants chose between different effectors (e.g., the two middle and index fingers) to make a button press. The authors manipulated the amount of information that was available before the response cue, by precueing response hand (left or right), response finger (middle or index), or neither. Electromyographic (EMG) activity was recorded from the prime movers of the response fingers. This enabled the authors to divide the overall RT into a PMT interval (from go signal to EMG onset) and an MT interval (from EMG onset to button press). They found that reducing the number of response alternatives shortened PMTs as well as MTs (Possamaĩ et al., 2002), suggesting that precueing does not only affect response-selection (cf. Goodman and Kelso, 1980), but also motor processing (cf. Rosenbaum, 1980).

### RT fractionation applied to speech-production research

The idea of subdividing the overall time that is needed to complete a correct response after a mental operation has also been used in psycholinguistics. For example, Hennessey and Kirsner (1999) divided the overall time that is needed to read aloud a written word vs. naming aloud a picture, into a response latency (i.e., pre-articulation) and a response duration (i.e., during-articulation) interval. Typically, and in this study as well, response latencies are shorter for word reading than for picture naming (Cattell, 1885; Fraisse, 1969). In addition to this well-known effect, however, the authors found that response durations were longer for word naming (for low-frequency items only). On the basis of this trade-off, they reasoned that naming of a written word can be initiated on the basis of partial information (e.g., the phonology of the word's beginning), resulting in faster RTs. As a consequence of this early initiation, the remainder of cognitive processing (e.g., the rest of the phonology) has to be carried out during response execution. This, in turn, results in longer response durations (for a similar account, see Damian, 2003; but see also Rastle et al., 2000).

Kello et al. (2000) and Damian (2003) used the same sub-division. They did so in order to investigate whether the effect of a Stroop manipulation cascades down to articulatory processing. Kello et al. (2000) found that when participants were put under high time pressure, they demonstrated a Stroop effect on response durations. In contrast, Damian (2003) did not replicate this effect.

An even more fine-grained method was used by Kawamoto et al. (1999), who sub-divided response durations of monosyllabic words into yet two different components: the duration of the initial consonants and the duration of the subsequent rime (the vowel following the consonants). They investigated the effect of word frequency on both dependent variables and found that initial-phoneme durations, but not rime durations, were shorter for high-frequency words compared to low-frequency words. From these findings the authors concluded that the criterion to initiate pronunciation is based on the initial phoneme and not on the whole word. This result challenges the assumption that articulation is initiated only after phonological encoding has been completed (Levelt et al., 1999).

**Figure 1:**
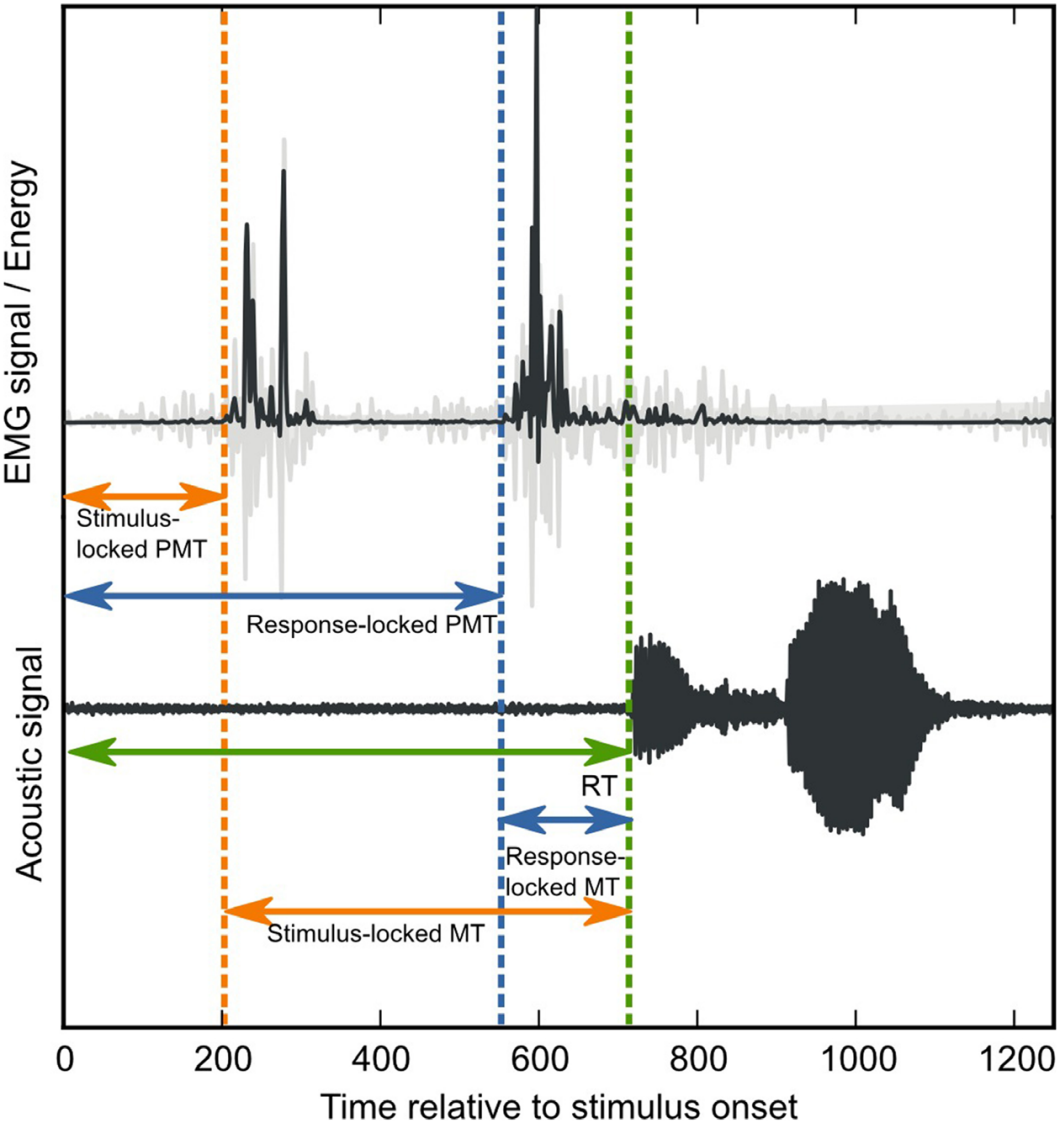
**Verbal-RT fractionation**. Verbal response time (green arrow) is divided into a PMT interval, between stimulus onset and EMG onset, and an MT interval, between EMG onset and vocal-response onset. The green-dotted line indicates vocal-response onset. In order to determine EMG onsets, EMG signals (displayed in light gray) were first transformed into the TKE domain (displayed in dark gray, see also Methods). Next, EMG onsets were determined by the stimulus-locked onset-detection method (orange dotted lines) and the voice-locked onset-detection method (blue dotted lines). The effect of both methods on the PMT and MT intervals, is indicated by the length of the orange vs. the blue arrows.

We recently supplemented the existing collection of research methods by adapting the above-described RT-fractionation procedure from manual-decision-making tasks to speech-production tasks (see also Towne and Crary, 1988). To do so, we simultaneously recorded standard acoustic voice signals as well as EMG activity from several lip muscles involved in speech articulation, while participants carried out picture naming and word reading tasks (Riès et al., 2012, 2014). We defined verbal RT as the delay between stimulus presentation and the onset of the vocal response (Oldfield, 1971). Next, determining the onset of EMG activity enabled us to divide the stimulus-response (SR) interval into a PMT interval (from stimulus onset to EMG activity) and an MT interval (from EMG activity to verbal response, see Figure 1). Upon reanalysis of our previous data, we found that the difference in reading vs. naming times can be solely attributed to the PMT interval, and not to the MT interval (Riès et al., 2014 and see below).

Our previous RT-fractionation research revealed that defining the point of RT fractionation on the basis of EMG bursts is not straightforward in speech-production tasks. This difficulty is the focus of the current paper.

### Defining the point of fractionation

There is an important difference between the previously described button-press tasks, in which RT fractionation has been repeatedly used, and speech-production tasks. A button press results from the activation of muscle fibers in the prime mover of the response finger. Therefore, EMG activity recorded from accordingly placed electrodes is necessarily coupled to the overt manual response. Articulating a verbal message, on the other hand, is a more complex action. It involves a fine-grained coordination between effectors, and does not only require lip-muscle movements, but also vibrations of the vocal folds (Browman and Goldstein, 1992). Because these are different effectors, voicing (i.e., vocal-fold vibration) can occur without any preceding lip-muscle EMG activity, and lip-muscle EMG activity can occur without any subsequent voicing. In addition, because of the very high number of muscles and effectors involved in articulation, the coordination of speech movement involves multiple degrees of movement freedom (Gracco, 1988).

**Figure 2:**
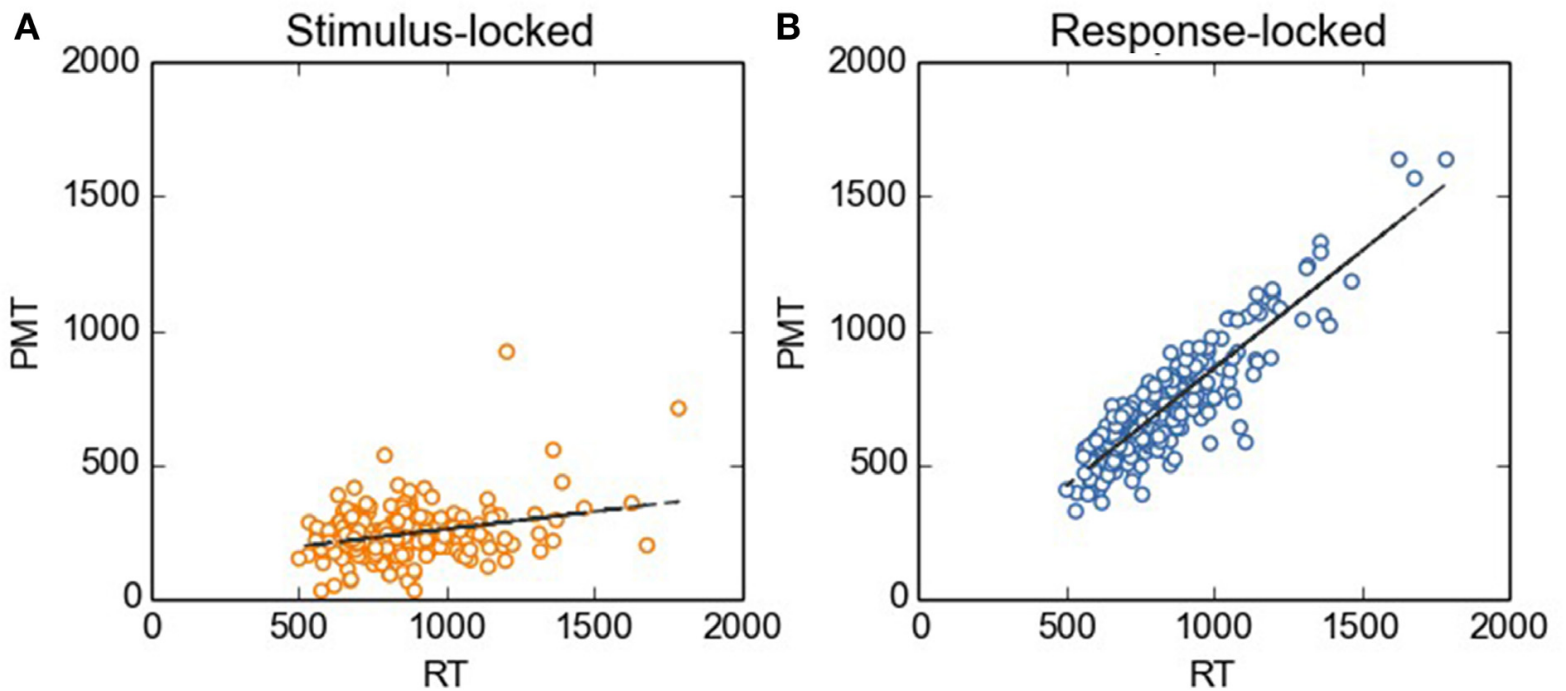
**Stimulus-locked (A) and response-locked (B) PMT as a function of RT**. Before carrying out the regression analysis described in the main text, we removed the between-subjects variability by using the method as described by Cousineau (2005). For the sake of comparison, the scale on the *y* axis is kept constant across Figures.

In our previous research (Riès et al., 2012, 2014), we observed that facial EMG, unlike manual EMG, often contains several bursts of EMG activity within the interval of interest (see Figure 1, see also Supplementary Material). This introduces an uncertainty when choosing the fractionation point for the SR interval. On the one hand, one could reason that RTs should be fractionated on the basis of the first burst of EMG activity after stimulus onset. After all, only the interval prior to this event reflects pure pre-motor processing. We refer to this approach as the *stimulus-locked* approach (see Figure 1 orange dotted line). On the other hand, one could argue that when the purpose is to investigate whether cognitive manipulations change the articulation of the verbal response, RT fractionation should be carried out on the basis of the burst of EMG activity that is coupled to the vocal response. This is because only these bursts of EMG activity can safely be assumed to reflect articulatory processing, whereas earlier bursts could reflect anything (e.g., a startling response, an aspecific preparation to speak, etc.). We refer to this approach as the *response-locked* approach (see Figure 2, blue dotted line).

The operationalization of the point of fractionation directly influences the duration of PMT and MT: If the point of fractionation is early in the SR interval, PMT will be short, whereas MT will be long. The reverse is true if the point of fractionation is only later in the SR interval. In turn, these durations influence how much of the task or manipulation effect, present on overall RTs, can be captured by either intervals. Thus, if an error in the onset-detection procedure makes that one of both intervals is “artificially” long, this interval is likely to falsely inherit part of the task or manipulation effect. Because this is a serious problem, in the current paper we investigate the influence of a methodological choice, that is, using the stimulus-locked and the response-locked onset-detection method, on task effects. The purpose is to make an informed decision about which of the two approaches is preferable.

### The current study

In the case of verbal responses, it is difficult to make inferences about the cognitive-processing stages that underlie an observed series of EMG bursts. For example, by merely looking at our EMG signals it is impossible to determine whether the early EMG bursts occurring shortly after stimulus onset represent some sort of aspecific preparation, breathing, or startle response, in which case they should not be used for RT fractionation, or whether they are an indispensable component of the articulatory process producing the subsequent vocal response, in which case they should be used for RT fractionation.

Yet, we do think it is possible to formulate a minimum set of objective criteria that a valid RT-fractionation method should meet given its stated purpose to distinguish cognitive from motor processing intervals. Firstly, the point on which RTs are fractionated should represent the burst of muscle activity that reflects the initiation of the articulation of the subsequent response. Therefore, valid fractionation points are expected to correlate strongly with verbal RTs. Secondly, the PMT interval should reflect cognitive processing stages such as response selection. Therefore, tasks that are known to tap into this processing stage should at least have an effect on the duration of the PMT interval, regardless of whether this effect may additionally cascade down to the MT interval.

In order to compare the stimulus-locked and the response-locked approach on the basis of these two criteria, we analyzed the data from a Stroop task (Stroop, 1935). We recorded vocal responses and EMG activity from several lip muscles and fractionated RTs twice: once by using the stimulus-locked approach and once by using the response-locked approach. Our reason for choosing the Stroop task was twofold: Firstly, the Stroop task is known to have a strong effect on RTs. We reasoned that the stronger the task effect on RTs would be, the easier it would be to demonstrate the previously mentioned problem of false inheritance. Secondly, even though the articulatory locus of the Stroop effect is debated (Kello et al., 2000; Damian, 2003), it is established that the Stroop effect should at least have a substantial cognitive, response-selection component (e.g., Logan and Zbrodoff, 1998; Damian, 2003; Damian and Freeman, 2008; Zurrón et al., 2013). This should result in a Stroop effect on PMTs.

## Methods

### Participants

Eighteen native French speakers with normal or corrected-to-normal vision participated in the experiment (mean age: 20.6, *SD* = 1.5 years). The data of seven participants were excluded from the analysis due to over-noisy EMG recordings, thus leaving 11 participants for the analysis.

### Stimuli

We carried out a classic verbal Stroop experiment (Stroop, 1935). Ink color was blue (requiring, in French, the response “bleu”), brown (“marron”) or orange (“orange”). These colors were chosen because their names started with labial phonemes. Letter strings were words that were congruent with the to-be-named color (e.g., “bleu” if the ink color was blue), incongruent with the to-be-named color (e.g., “marron” if the ink color was blue), or neutral (letter string “iiiii”). In the incongruent condition, for a given ink color the interfering word was fixed (e.g., for the color blue the interfering word was always “marron,” etc.), resulting in nine possible color-word combinations (three per condition).

### Procedure

Each trial consisted of the following sequence: (1) a fixation point (“+” sign) of which the duration varied randomly between 500 and 1000 ms, (2) the letter string, presented until the participant responded or a 1500 ms deadline was reached, and (3) a blank screen for 2000 ms. Participants were instructed to name the ink color of a visually presented letter string as fast and accurately as possible. The experiment consisted of one block in which all nine stimuli were presented 15 times, resulting in a total of 135 trials. Trial order was randomized, and kept constant across participants.

### EMG and voice recordings

Voice and EMG signals were simultaneously recorded by the same device (Keithley Instruments, Inc.). The acoustic signal was recorded at 28,000 Hz. Bipolar montages of 6 mm-diameter Ag/AgCl surface electrodes (Grass Technologies, Inc.) were used to record EMG activity from four facial muscles: *levator labii superioris*. *risorius*. *orbicularis oris*, and *depressor labii inferioris*. Sampling rate for EMG recordings was 2000 Hz and the ground electrode was placed over the left collarbone. This bipolar montage can be readily performed with any commonly-used EEG recording system as long as the electrodes are small enough to allow two of them to be placed on each of the muscles of interest. Off-line low-pass filter were applied to the EMG and acoustic signal (300 Hz, 2680 Hz, respectively).

### EMG-onset detection

As mentioned above, when multiple bursts of EMG activity are present within the SR interval, the point on which overall RTs are fractionated into PMT and MT can be defined in at least two possible ways: (1) on the basis of the first burst of EMG activity after stimulus onset (the stimulus-locked approach), or (2) on the basis of the EMG activity closest to the sound onset of the verbal response (the response-locked approach). Depending on the definition, the experimenter should search for bursts of EMG onsets from stimulus onset onwards, or around the verbal response, respectively (see Figure 1, orange and blue dotted lines, respectively). In order to make an informed comparison between both procedures, we determined EMG onsets that were used as the point of fractionation according to both definitions separately. We did this for one facial muscle, the *depressor labii inferioris*.

Stimulus-locked EMG onsets were detected as follows: First, to facilitate EMG-onset detection, we applied the Teager-Kaiser Energy operation (TKEO) to the EMG signal. By doing this, abrupt changes in amplitude as well as in frequency are measured. Previous studies have shown that this operation greatly improves the signal-to-noise ratio of EMG signals (Li et al., 2007; Solnik et al., 2008, 2010; Lauer and Prosser, 2009). The TKEO Φ on a given sample is defined as:

$$\Phi [i]=X_{i}^{2}-\left(X_{\left\{i+1\right\}}X_{\left\{i-1\right\}}\right)$$

where *X* is the EMG amplitude for sample *i*. Phrased simply, this operation indicates how much the amplitude of a given sample differs from the amplitude of the previous (*i* – 1) and the subsequent (*i* + 1) sample.

Next, a logistic signal (i.e., 0 vs. 1) was obtained by thresholding the TKE-processed signal. Then, to minimize the chance of false alarms, the logistic signal was low-pass filtered with a moving window. Finally, this smoothed signal was thresholded again. The first sample that exceeded this threshold was the point of fractionation as detected by the stimulus-locked approach.

Response-locked fractionation points were determined by investigating EMG signals in a backwards manner, starting 100 ms after voice onset^1^. To this end, we used a semi-automatized procedure of which the first steps were identical to the stimulus-locked approach. The only differences were that, in order to look back in time, we applied the above-described algorithm to the reversed EMG signal, and the final thresholding stage was set such that the algorithm searched for the first sample of which the smoothed signal was lower than a certain threshold.

All automatically detected onsets were visually checked and, if necessary, manually edited by an expert who was blind to the condition of the trials. The package that we used for both procedures is available from the first author's website (https://github.com/lvanderlinden/OnsetDetective).

### RT fractionation

Once the relevant EMG bursts were identified, we defined three dependent variables per trial: the standard vocal RT, the PMT, and the MT. We did this separately on the basis of stimulus-locked and response-locked EMG bursts. Note that, by construction, RT = PMT + MT. Analyses usually performed on RTs, such as analyses of variance or regressions, can also be performed on PMTs and MTs.

## Results

The purpose of the current paper was to investigate different methods for detecting EMG bursts, a stimulus-locked vs. a response-locked approach, to assess which one was more suitable to distinguish pre-motor from motor times (PMT vs. MT). Because on some trials multiple EMG bursts were not present or not clearly dissociable (see heatmaps Supplementary Material) these trials were not useful for investigating the pure effect of stimulus- vs. response-locked RT fractionation. After all, on these trials both onset-detection methods may detect the same burst of activity. Therefore, as a first step we only selected those trials on which the difference between both approaches was clear, based on visual inspection of the data. This was the case for 25% of the trials (congruent condition: 69 trials, neutral condition: 68 trials, incongruent condition: 105 trials). Additionally, we excluded trials on which the EMG onset occurred after vocal-response onset^2^. Finally, we excluded the data from two participants for whom some conditions did not contain any trials after applying these strict exclusion criteria. This resulted in a selective data set containing 16% of the total number of trials (238 out of 1485 trials). The figures and the statistics reported in the text below are based on this data set and indicate the pure effect of stimulus- vs. response-locked RT fractionation.

As a next step, we investigated to what extent this subset of extreme trials influences the general conclusions that are drawn over a broader data set, including trials on which the distinction between stimulus- and response-locked fractionation is absent or less evident. For this wider selection, we only discarded trials on which EMG onset detection was ambiguous because (1) participants showed bursts of EMG activity in the baseline period prior to the stimulus onset, (2) signal-to-noise ratio during the SR interval was too low to detect transitions from resting state to muscle activity, (3) voice production other than the desired response (e.g., hesitations) influenced the acoustic signal, or (4) response-locked EMG activity occurred after voice onset. We ended up with 51% of trials on which stimulus- and response-locked EMG bursts, if present, could be detected relatively unambiguously^3^ (congruent condition: 178 trials, neutral condition: 186 trials, incongruent condition: 210 trials). Of course, such a rejection rate would have been suboptimal if the main purpose of our analyses was to investigate a task effect (here, Stroop condition) on a set of dependent variables. However, the purpose of the current paper was to demonstrate the influence of a methodological choice (stimulus- vs. response-locked onset detection) on these task effects. The aim of our conservative exclusion criteria was to exclude the possibility that any observed differences between both approaches were due to general issues with onset detection (regardless of whether it is carried out in a stimulus- or voice-locked manner). The analyses carried out over the resulting broader data set are reported in brackets and indicate to what extent the influence of extreme differences between stimulus- and response-locked fractionation influence the conclusions drawn from the entire data set.

### Effect of the two approaches on MT and PMT intervals

When applying the stimulus-locked approach, the EMG burst that was used for RT fractionation was typically found early in the SR interval, resulting in short PMTs (*M* = 245.31, *SE* = 17.56, for broader data set: *M* = 291.21, *SE* = 15.23), and long MTs (*M* = 605.14, *SE* = 13.00, for broader data set: *M* = 496.77, *SE* = 19.35). When applying the response-locked approach, the point of fractionation was typically found late in the SR interval, resulting in long PMTs (*M* = 720.44, *SE* = 14.51, for broader data set: *M* = 661.74, *SE* = 21.24) and short MTs (*M* = 130.01, *SE* = 16.44, broader data set: *M* = 126.24, *SE* = 15.83). The fact that PMT and MT durations are extremely dependent on the detection procedure already shows that it is important to select the appropriate procedure.

Firstly, we investigated to what extent stimulus- vs. response-locked points of fractionation correlated with RT. As aforementioned, our reasoning was as follows: The transition from PMT to MT should be the initiation of the articulation of the subsequent vocal response. Hence, the EMG bursts used as fractionation points should correlate with RTs. To test this prediction, we did linear-regression analyses with RT as the independent variable, and stimulus-locked and response-locked PMTs as the dependent variables. As can be seen in Figure 2, when RTs were divided on the basis of stimulus-locked EMG bursts, PMTs did not correlate strongly with RTs (*R* = 0.29, *R*^2^ = 0.08, *p* < 0.0001, broader data set: *R* = 0.17, *R*^2^ = 0.03, *p* < 0.0001). Instead, when RTs were divided on the basis of response-locked EMG bursts, we observed the expected pattern: RTs correlated more strongly with PMTs (*R* = 0.89, *R*^2^ = 0.79, *p* < 0.0001, broader data set: *r* = 0.87, *R*^2^ = 0.76, *p* < 0.0001).

Thus, later-occurring EMG bursts appear to be locked to the response onset whereas early EMG onsets are not. To corroborate this finding, we calculated the decimal logarithm of the ratio between the variances of MT and PMT (varMT/varPMT) for each participant, separately for both onset-detection approaches. These log-transformed ratios have been shown to be a good index of the strength of the relationship between the occurrence of the event of interest (here, the EMG onset) and the beginning of the stimulus vs. the beginning of the motor response (Commenges and Seal, 1985)^4^. A positive value is expected if the event is more strongly related to stimulus onset, whereas a negative value is expected if the event is more strongly related to response onset. A zero value is expected if the event is not strongly related to either.

As predicted, we found that for the response-locked approach, the log-transformed ratios were smaller than zero for all participants [*M* = −2.43, *SD* = 0.58, *t*_(8)_ = −11.80, *p* < 0.0001, for the broader data set: *t*_(10)_ = −13.30, *p* < 0.0001]. For the stimulus-locked approach, this was not the case. Instead, the log-transformed ratios were larger than zero for all participants [*M* = 1.78, *SD* = 1.04, *t*_(8)_ = 4.85, *p* = 0.001, for the broader data set: *t*_(10)_ = 4.18, *p* = 0.002]. These results indicate that only the response-locked approach yields EMG bursts that are locked to the response onset.

In sum, the results of our first set of analyses suggest that the response-locked approach better captures the intended point of RT fractionation than the stimulus-locked approach.

### Effect of the two approaches on the task effect on MT and PMT intervals

**Figure 3:**
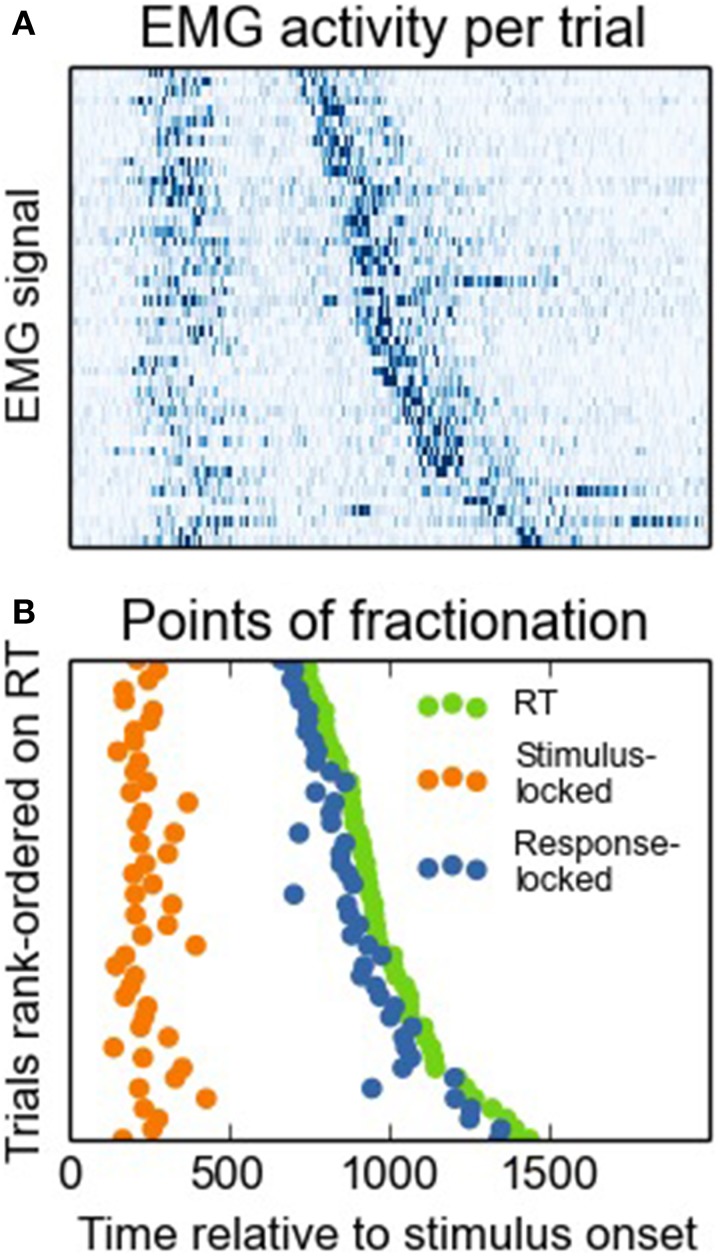
**Panel (A) depicts a heat map of normalized EMG signals per trial, rank-ordered on RTs**. High amplitudes correspond to dark shadings. Panel **(B)** depicts how we determined EMG onsets on the basis of these signals for the stimulus-locked vs. the response-locked approach.

**Figure 4:**
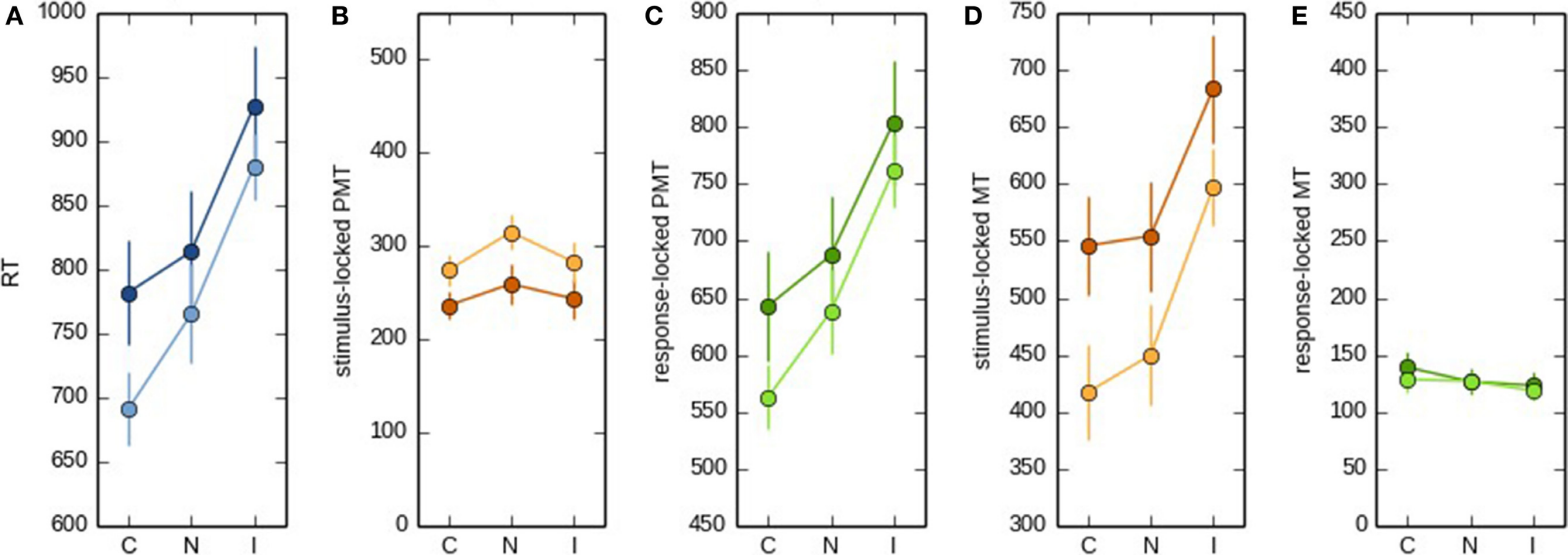
**RT (A), stimulus-locked (B) and response-locked PMT (C), and stimulus-locked (D) and response-locked MT (E) as a function of Stroop condition (congruent, neutral, or incongruent)**. The condition effects depicted by the dark lines are based on the selection of trials for which the difference between the two onset-detection approaches were maximal. The condition effects depicted by the light lines are based on the broader data set. For the sake of comparison, the scale on the *y* axis is kept constant across **(B,C)** and **(D,E)**.

Next, we investigated to what extent both onset-detection methods influenced task effects on PMT and MT intervals. Figure 3A shows a heat map of EMG activity for a participant for whom the interval between the different EMG bursts was large, and Figure 3B shows how this EMG activity resulted in stimulus- vs. response-locked points of RT fractionation. From looking at this Figure, it becomes apparent that for the stimulus-locked approach, MTs falsely inherit part of the task effect on overall RTs, even though these intervals might not reflect what we are aiming for, that is, articulatory processing of the eventual vocal response. To demonstrate this point empirically, we carried out analyses of variance with Stroop condition (congruent, neutral, or incongruent) as the within-subjects factor, and RT, as well as stimulus-locked and response-locked PMT and MT, as the dependent variables. The results are shown in Figure 4. Firstly, Stroop condition affected RTs such that participants were fastest on congruent trials, and slowest on incongruent trials [*F*_(2, 16)_ = 7.203, *p* = 0.006, broader data set: *F*_(2, 20)_ = 23.61, *p* < 0.0001, see Figure 4A; the difference in degrees of freedom is due to the broader data-set including data from two more participants, see above]. More importantly, Figure 4B shows that when stimulus-locked RT fractionation is used, the Stroop effect on PMTs is absent [*F*_(2, 16)_ = 0.95, *p* = 0.41; in the broader data set the effect is marginal but atypical and small in size, see Figure 4B, light lines, *F*_(2, 20)_ = 3.47, *p* = 0.051]. In contrast, there is a clear Stroop effect on MTs [*F*_(2, 16)_ = 7.12, *p* = 0.006; broader data set: *F*_(2, 20)_ = 14.59, *p* < 0.0001, see Figure 4C]. The response-locked method reveals the reverse pattern: a Stroop effect on PMTs [*F*_(2, 16)_ = 6.58, *p* = 0.008; broader data set: *F*_(2, 20)_ = 25.8, *p* < 0.0001, see Figure 4D] and no effect on MTs [*F*_(2, 16)_ = 1.23, *p* = 0.32; broader data set: *F*_(2, 20)_ = 0.835, *p* < 0.448, see Figure 4E].

In conclusion, again only the response-locked approach met our prediction, namely that a cognitive manipulation such as Stroop condition should at least show its classical effect on the PMT interval (see Figure 4C). Stimulus-locked RT fractionation failed to show the expected Stroop effect on the PMT interval (see Figure 4B). In other words, with respect to PMT, the stimulus-locked approach resulted in a Type II error, that is, the incorrect failure to reject the null hypothesis in favor of the alternative hypothesis.

Even more importantly, the stimulus-locked approach also increases the risk of making a Type I error, that is, an incorrect rejection of the null hypothesis. Because the PMT interval was depleted from an effect that should logically be there, the MT interval inherited part, if not all, of the task effect that should ideally be attributed to PMT (see Figure 4D). Although we cannot exclude the possibility that the Stroop effect has some effect on MTs, it is clear that the enormous effect on MTs observed with the stimulus-locked approach is at least largely artifactual.

## Discussion

We previously proposed a method to fractionate verbal RTs into a PMT (assumed to reflect cognitive processing) and an MT (assumed to reflect motor processing) interval on the basis of the onset of EMG activity as measured from several facial muscles involved in speech. However, we noticed that EMG signals from facial muscles often contain multiple bursts of activity. This observation forced us to make a decision about how to fractionate RTs: on the basis of the first EMG burst after stimulus onset (the stimulus-locked approach), or on the basis of the EMG burst that proceeds the vocal response (the response-locked approach). We fractionated verbal RTs of participants performing a Stroop task on the basis of both approaches, in order to investigate which of the two is preferable.

On the basis of our results, we conclude that there are both analytical-conceptual and methodological-statistical reasons to prefer the response-locked over the stimulus-locked approach. Firstly, the response-locked approach provides a better measure of what we intend to measure with our RT fractionation method: (1) an EMG burst that fractionates RTs on the basis of muscle activity that reflects the initiation of the articulation of a verbal response, and (2) a resulting PMT (assumed to reflect cognitive processing) that is indeed sensitive to a cognitive manipulation. The stimulus-approach did not show these two features. Secondly, our results demonstrated that the stimulus-locked approach attenuated the expected task effect on PMT, whereas it presumably overestimated the task effect on MT. The response-locked approach does not carry these risks. Therefore, in conclusion, we believe that when using RT fractionation in speech-production research, EMG onsets should be detected in a response-locked manner.

### Inter-individual variability

Our results showed a large inter-individual variability in the distribution of EMG activity throughout the SR interval. This suggests that articulatory coordination varies largely across speakers. Some participants showed clear isolated stimulus-locked EMG bursts (e.g., participant 1, see Supplementary Material). For others, the distinction between both onset-detection approaches was less clear (e.g., participant 11).

In the current experiment, there were only three possible first phonemes (/b/, /m/, /o/), and all first phonemes were labial. While we thought this would ease EMG detection, this may have enabled participants to prepare their articulation better. Indeed, manner of articulation has been shown to have a significant effect on acoustic latencies in speeded naming tasks (Kawamoto et al., 1998; Rastle et al., 2005). Some participants may have used the repetition of the type of first phoneme more than others. This inter-individual variation in articulatory preparation is worth exploring in further studies.

### Impact of our results on the electroencephalographic investigation of language production

**Figure 5:**
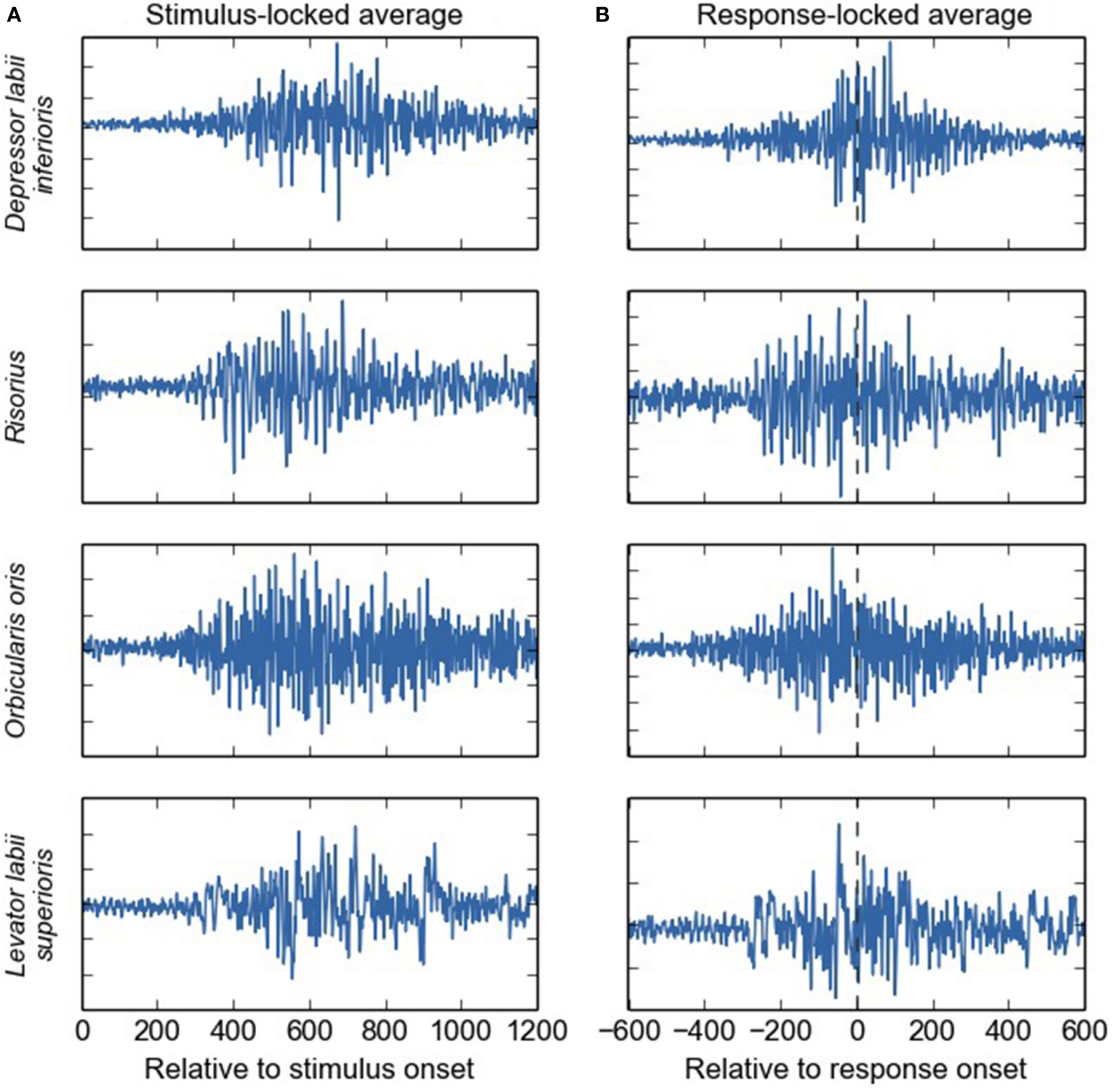
**Average EMG waveforms for four different facial muscles (in arbitrary units), event-locked on the stimulus (A) and on the response onset (B)**. Substantial EMG activity is present during a large part of the stimulus-response time-window, and not only shortly around the response event. We deliberately chose to average raw, instead of rectified, EMG signals, even though this will cause anti-phase activities to cancel out. We reasoned that this may give the most pure information on how EMG activity may impact scalp-recording averages performed over (unrectified) EEG signals. It is of note that in contrast to the rest of the paper, here, the terms “stimulus-locked” and “response-locked” refer to the event that is used to align the single-trial EMG signals on, rather than to refer to the onset-detection procedure.

The current results may also be relevant for electroencephalographic (EEG) studies of speech and language production, in which facial EMG activity is a major concern (for a review, see Ganushchak et al., 2011). Facial EMG activity is much larger than EEG activity and thus heavily contaminates the signal of interest (Morrell et al., 1971; Brooker and Donald, 1980; Friedman and Thayer, 1991; Goncharova et al., 2003; De Vos et al., 2010). One of the commonly used strategies to remove this EMG activity is to fractionate the verbal RT arbitrarily (e.g., at 600 ms post-stimulus or 100 pre-response) and to discard the period thought to be contaminated by EMG activity. Some features of the current results challenge this assumption by showing that facial EMG activity can occur much earlier in time (i.e., earlier than 300 ms post-stimulus, see Figure 1 and Supplementary Material), even though this EMG might not be functionally linked to articulation. The average waveforms in Figure 5 demonstrate that substantial EMG activity is present during a large part of the SR interval. This poses a problem for the common approach toward contamination of EEG by EMG, and suggests that the issue should be considered in more detail (see De Vos et al., 2010 for a possible solution).

### Future directions

Our current analyses were set up to test the validity of stimulus- vs. response-locked EMG detection, and not to investigate serial vs. cascaded processing in a verbal Stroop task. Therefore, and because of the general problems with drawing firm conclusions from null results (here, the absence of Stroop effects in MT), we are reluctant to make any claims about this issue in the current paper. However, provided that EMG onsets are detected using the response-locked approach, we think that verbal-RT fractionation is a valuable tool for investigating serial vs. cascaded processing in future research. We briefly discuss three research questions that could be well addressed by this method. Our suggestions have in common that previous research has already suggested that the flow of information in the proposed paradigms is cascaded.

Firstly, as mentioned previously, providing participants with advance information about the number of possible response alternatives reduces manual RTs (as well as PMTs and MTs, Possamaĩ et al., 2002). Analogously, advance information also facilitates word preparation. For example, Meyer (1990) carried out an implicit priming task, using a paired-associate learning paradigm. In a first phase, participants memorized blocks of word pairs, of which the first word functioned as a prompt, and the second one functioned as the response word. After the learning phase, participants performed experimental blocks. In these blocks, only the prompt word was presented, and participants had to name the corresponding response word as fast as possible. Importantly, there were two different types of experimental blocks: homogeneous blocks, in which all response words started with the same syllable, and heterogeneous blocks, in which response words were unrelated in form. As predicted, advance information about the first syllable shortened RTs. Although the author interpreted this in terms of phonological encoding, she acknowledged that motor preparation may also contribute: “When the response words shared the first syllable, the subjects could bring their speech organs into an optimal starting position to utter the response word” (Meyer, 1990, p. 540). To our knowledge, the latter possibility has received surprisingly little attention afterwards, even though the analogy with manual response selection suggests that a dual locus of the priming effect is plausible (Possamaĩ et al., 2002). Therefore, fractionating the effect into PMT and MT components would be a logical next step, especially because the implicit-priming paradigm is widely used to address issues of phonological encoding, without further consideration of the alternative motor-preparation interpretation (e.g., Damian and Bowers, 2003; Alario et al., 2007; Rastle et al., 2011).

Secondly, facial EMG measurements and RT fractionation could be used in order to investigate the links between speech perception and speech production. Several researchers have argued that perceiving speech activates the motor system (Yuen et al., 2010; but see also McGettigan et al., 2010). Yuen et al. (2010), for example, asked participants to produce target syllables while simultaneously hearing distractor sounds. Distractors could be congruent (the same syllable), or incongruent (a rhyming syllable with a different first phoneme) with the target. Interestingly, incongruent distractors changed the articulatory trajectories of the spoken syllable, such that it contained traces of the distractor. From this finding, the authors concluded that speech perception indeed automatically activates motor programs. Future research could use facial EMG measurements and verbal-RT fractionation in order to investigate whether incongruent sounds interfere with the coordination between articulatory effectors, whereas congruent sounds facilitate articulation. More precisely, the prediction would be that incongruent sounds would lengthen MTs, whereas congruent sounds would shorten MTs.

Finally, facial EMG measurements and RT fractionation could be employed to extend the few previous studies that investigated cascading between cognitive and motor processing in speech production. As mentioned in the introduction, Goldrick and Blumstein (2006) employed a tongue-twister paradigm and showed that erroneous responses contained traces of the intended target, as if two responses had been prepared simultaneously. Following this logic, solving the competition between two simultaneously prepared phonemes may take longer for erroneous responses than for correct responses, thereby lengthening both PMTs and MTs (for similar results in manual responses, see Allain et al., 2004). Verbal-RT fractionation could directly test this hypothesis, and examine whether the conclusions are generalizable to other speech features than voicing.

We conclude that, with the proper methodological precautions, combining the analysis of articulatory gestures with mental chronometry may be a valuable method. Using MTs as a dependent variable could help combining previous knowledge from psycholinguistic and motor-control research into one integrated approach to understanding speech production (Hickok, 2014).

### Conflict of interest statement

The authors declare that the research was conducted in the absence of any commercial or financial relationships that could be construed as a potential conflict of interest.

## Footnotes

^1^In speech-production tasks, EMG activity can occur after voice onset. This is because EMG activities recorded from facial muscles and voice onsets correspond to the actions of different vocal-tract effectors (protrusion and/or aperture of the lips and glottal aperture). To allow for such “inversions” to be captured by our EMG onset-detection method, we initiate the response-locked approach 100 ms after voice onset, instead of at voice onset.

^2^In the case of inversions, the “RT = PMT + MT” principle yields negative MTs. Because the interpretation of these MTs is more ambiguous than the interpretation of MTs with a positive value, for the current analysis we excluded trials on which an inversion occurred. However, it is of note that the here-reported results do not change when inversions are included.

^3^We note that if a stimulus-locked EMG onset could not be detected (e.g., because of a too-low SNR, or because there was activity already during baseline), this trial was not included in the response-locked EMG onset detection. This was done for the purpose of the current study and it is possible that more trials would have been spared if only the response-locked approach had been used.

^4^The decimal logarithm of variances is widely used in neurophysiological studies (Requin et al., 1988; Mouret and Hasbroucq, 2000). The reason for using the logarithm of the varMT/varPMT ratio, instead of the ratio itself, is that the variance ratio is not a symmetrical function with respect to zero. More precisely, if varMT is smaller than varPMT, ratios will vary between 0 and 1, whereas if varMT is larger than varPMT, ratios will vary between 1 and infinity. In contrast the decimal logarithm is symmetrical around zero (Requin et al., 1988; Mouret and Hasbroucq, 2000). This facilitates the comparing the two possible directions of the ratio.
