# Supplementary figures and images for: A comparison of two procedures for verbal response time fractionation
Source: Front Psychol. 2014 Oct 24;5:1213. doi: 10.3389/fpsyg.2014.01213 (PMC4208410; doi:10.3389/fpsyg.2014.01213)

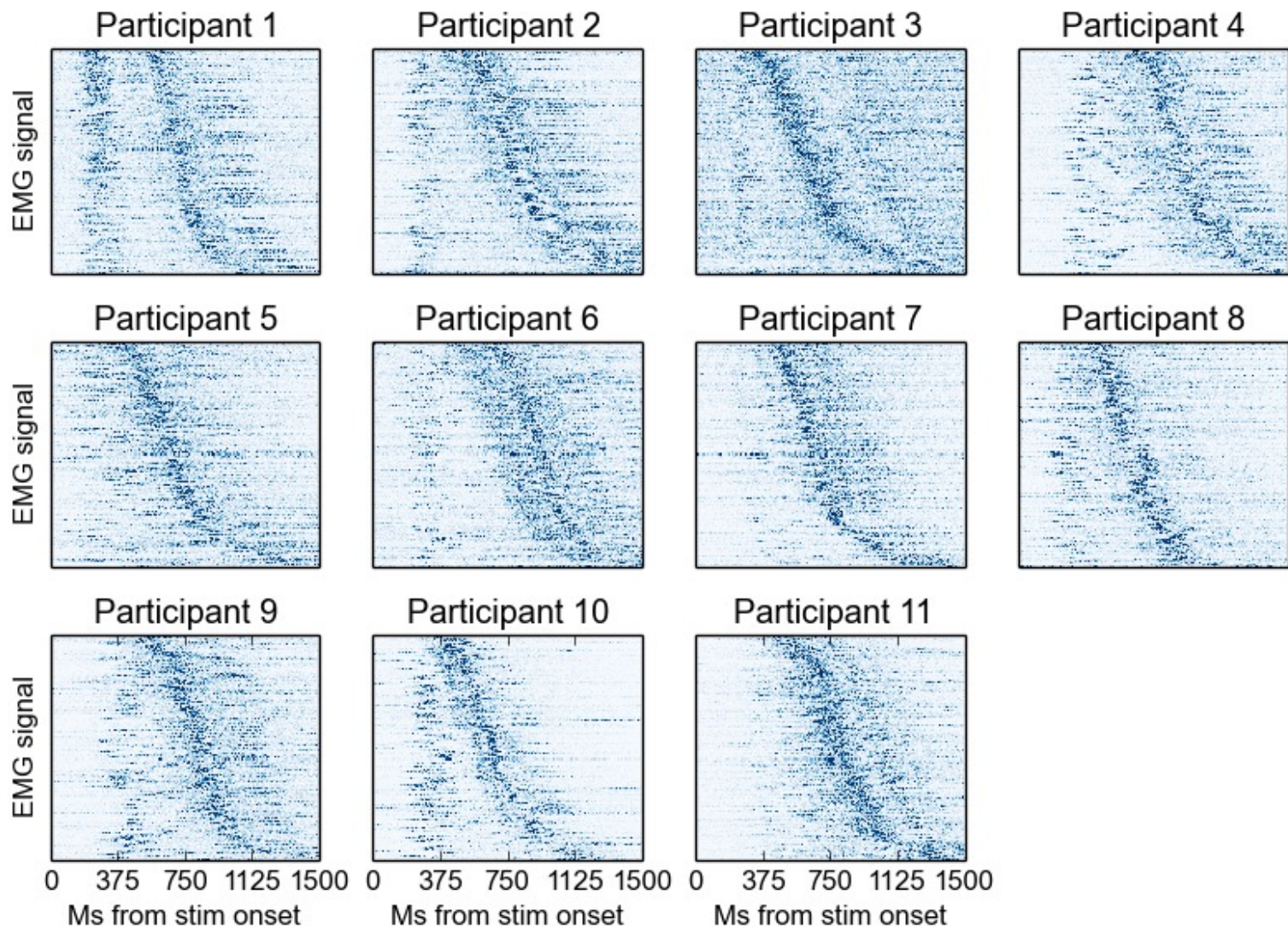

Supplement: Supplementary file 1 [file Presentation1.PDF]
